# Comparative Chemical Composition and Antioxidant Properties of the Essential Oils and Aromatic Water from *Teucrium persicum* Boiss

**Published:** 2012

**Authors:** Abdolhossein Miri, Hamid Reza Monsef-Esfahani, Mohsen Amini, Yaghoub Amanzadeh, Abbas Hadjiakhoondi, Reza Hajiaghaee, Atefeh Ebrahimi

**Affiliations:** a *Department of Pharmacognosy, Faculty of Pharmacy, Zabol University of Medical Sciences, Zabol, Iran.*; b *Department of Pharmacognosy, Faculty of Pharmacy, Tehran University of Medical Sciences, Tehran, Iran. *; c *Department of Medicinal Chemistry, Faculty of Pharmacy, Tehran University of Medical Sciences, Tehran, Iran.*; d *Medicinal Plants Research Center, Tehran University of Medical Sciences, P.O. Box: 14155-6451, Tehran, Iran. *; e *Department of Pharmacognosy, Institute of Medicinal Plants, ACECR, Karaj, Iran. *; f *Department of Biology, Faculty of Sciences, Central Herbarium of Tehran University, University of Tehran, Tehran, Iran.*

**Keywords:** Teucrium persicum Boiss, Antioxidant properties, Arraq, Essential oils, Aromatic water

## Abstract

The essential oils and aromatic water, known as Arak in traditional Iranian medicine, comes from the aerial part of Teucrium persicum Boiss., which is grown in Fars Province located in Iran. The samples were collected in summer and the oils and aromatic water were obtained through steam distillation. The chemical composition of the oils was analyzed using GC-MS. An analysis of the chemical profile of the isolated oils revealed the presence of more than 80 compounds, mainly oxygenated monoterpenes and sesquiterpene hydrocarbons. The principal components of essential oil were α-cadinene (9.7%), 1,4-cadinadiene (9.2%) and *α*-terpinyl acetate (7.9%). The major constituents in the Arak were determined to be linalool (10.4%), *α*-cadinene (7.5%) and *γ*-terpineol (7.3%). Most of the compounds identified from different oils were similar, but their amounts differed. The oil revealed a higher content of total phenolics than the Arak (1.71 ± 0.12 mg GAE/g DW and 1.36 ± 0.11 mg GAE/g DW, respectively). The antioxidant activity of the oils was calculated by using a ferric reducing antioxidant power assay (FRAP), DPPH radical scavenging activity, and a reducing power assay (RP). The FRAP value points to a considerably higher reducing power of essential oil (220 ± 7.2 µmol Fe^2+^/g DW) compared to that of Arak (113 ± 5.4 µmol Fe^2+^/g DW). Essential oil exhibited higher radical scavenging potential (IC_50_ = 0.29 mg/mL) than Arak (IC_50_ = 4.19 mg/mL). The reducing power of essential oil (51.7 ± 4.3 µg BHA/g DW) was higher than that of Arak (34.1 ± 2.7 µg BHA/g DW). The studied essential oils showed good antioxidant activities, which were higher than those of Arak.

## Introduction

Essential oils have long been used as flavoring agents and ingredients for several commercial products as well as folk medicine (1). These compounds are a complex mixture of chemicals with considerable activities.

The importance of essential oils is due to the increasing demand for food, cosmetics and pharmaceutical industries, so it is necessary to study yields, chemical profiles and biological activities of essential oils.

The genus *Teucrium *(Labiatae) includes about 300 species worldwide. This genus is distributed mainly throughout the Northern Hemisphere, in Central and South America, Southeast Asia, and the Mediterranean region (2, 3). *T. persicum*, an endemic plant in Iran, is distributed in Fars Province, around Lar Mountains (4). Natives named the plant *marv e talkh* and used it to treat headaches and abdominal pains such as colitis. In some communities, *Teucrium* has been used to treat diseases such as diabetes, obesity, hyperlipidemia, inflammation, and rheumatoid (5-7). The properties of this genus, such as antibacterial, antinociceptive, antioxidant, anticancer, tonic, and diaphoretic effects have been considered (2, 3, 5, 7-11). Many studies have been conducted to find an effective natural source of antioxidant compounds because of the complications from oxidative agents, such as autoimmune diseases, inflammation, cardiovascular disorders, arthritis, liver diseases, arthrosclerosis, cancer, and aging (7, 12).

In this context, several studies were carried out on the chemical composition and antioxidant properties of the essential oils of *Teucrium* species. Many of these oils showed effective antioxidant activity (4, 13-17). Hydrolats were used for their organoleptic, biological, and allelopathic properties in foods, cosmetics and agricultural products. Orange and rose aromatic water have been traditionally used in the Mediterranean region for skin care and the preparation of cakes and beverages (18). In Iranian folk medicine, the hydrolat which is called *Arak0\æræq\* has been used in traditional medicine.

This study evaluates the chemical composition, antioxidant activity and yield of essential oils and *Arak* of *T. persicum*. As far as we know, this work is the first report on the chemical composition of *Arak* and the antioxidant activities of this species.

## Experimental

*Plant material*

Aerial parts of the plant were collected in September 2009 at an altitude of 1,200 meters on Lar Mountain near Barak Village in Iran. Plants were dried in the shade at room temperature. A voucher specimen (No. 397) has been deposited at the Central Herbarium of Medicinal Plants (ACECR) in Iran.

*Essential oil isolation*

The air-dried aerial parts of the plant were subjected to steam distillation for 3 h using a Clevenger apparatus. The oil was dried in anhydrous sodium sulphate and stored at 4°C in tightly-closed dark container.

*Aromatic water extraction procedure*

Herbal Water (aromatic water) which is called Arak in some countries such as Iran, Afghanistan, and Arabic countries is steam distilled from Plants. Arak contains the essential oil of the plant in a natural water base.

Aromatic water, come out from Clevenger apparatus, was decanted three times with diethyl ether and then the diethyl ether was evaporated at room temperature. It was dried by anhydrous sodium sulphate and stored at 4°C until GC/MS analyses process.

*Gas chromatography-mass spectrometry analysis*

GC–MS analyses were performed using a Hewlett-Packard 5973–6890 system operating in EI mode (70 ev) equipped with a HP-5MS column (30 m × 0.25 mm, film thickness of 0.25 μm) and a split injector (290°C). The split ratio was 1:10. The column temperature program was 50°C (5 min) to 240°C at a rate of 3°C/min. The injection volume was one µL and the detector temperature was 290°C. The flow rate of helium as a carrier gas was 0.8 mL/min.

The identification of the constituents of the oils was performed through the comparison of their retention indices with the NIST database and literature data, as well as using the comparison of their mass spectra with the Wiley7n.l Mass spectral database and those described by Adams (19, 20).

Quantitative data was calculated using electronic integration from the FID area data without the use of correction factors.

*Antioxidant activity*

Antioxidant power was measured through three *in-vitro* methods including: 2, 2-Diphenyl-1-picrylhydrazyl (DPPH) assay; ferric reducing antioxidant power (FRAP); and reducing power (RP).

*DPPH assay*

The DPPH antioxidant assay is based on the spectrophotometric method according to Tofighi *et* *al*. (2009). In this test, the sample was reacted in dark at the room temperature for 30 min, with a certain concentration of methanolic DPPH. The difference in the absorbance between the initial purple-colored stable radical DPPH and the terminal yellow-colored DPPHH, at 517 nm, was determined to be antioxidant activity.

The scavenging percentage of the DPPH radical was calculated through the following formula (21-23): DPPH scavenging activity (%) = 100 × (1- (Abs sample/Abs initial DPPH)). IC_50_ (concentration of samples providing 50% scavenging) were calculated from the curve scavenging percentage against the oil concentration. The lower IC_50_ value means a higher antioxidant power. Butyl hydroxyanisol (BHA) and *α*-tocopherol were used as controls.

*Ferric reducing antioxidant power assay (FRAP)*

The FRAP assay is based on the ability of a sample to reduce Fe^3+^ in a Tripyridyltriazine (TPTZ) solution to Fe^2+^ and create the blue-colored complex Fe^2+^ – TPTZ.

Increased concentrations of the above complex means an increased FRAP value. The ability of the reducing power of the test sample was determined by using a spectrophotometer at 593 nm. This assay was done according to Benzie and Strain (1996) (24, 25).

*Reducing power assay*

The reducing power of the oils was measured according to the method used by Hinneburg *et al*. (2006). This method is based on the abilities of a sample to reduce ferricyanide to ferrocyanide and produce a Prussian blue-colored complex (Fe^3+^)_4_[Fe^2+^ (CN^-^) _6_]_3_ that is detectable. Its absorbance was measured at 700 nm. Increased absorbance of the reaction mixture interprets as an increase in reducing the power of sample. BHA was the control (22, 26, 27).

*Total phenolic content (TPC)*

The TPC of oils was determined according to the method using by Ghafar *et al.* (2010). This method is set up for the oxidation of phenolics using a molybdotungstate in a Folin-Ciocalteu reagent yielding a green-colored product with λ_max_ 745-750 nm. This assay lacks specifics for phenols and its reagents may react with disturbed compounds such as sugars, organic acids and aromatic amines and therefore, providing a concentration higher than real TPC (28). The recommended method for the determination of TPC is based on the spectrophotometric method (22, 28). Gallic acid was used as the standard for the calibration curve (20-200 mg/L, y = 0.003 x - 0.027, R^2^ = 0.991). TPC was expressed as mg Gallic acid equivalents (GAE) per g of DW.

*Statistical analysis*

Data was expressed as mean ± SD and all tests were repeated three times. Statistical analyses such as plots, student’s t-test, and p-value were done using Excel 2007.

## Results and Discussion

*Productivity and physical properties*

All oils of the aerial part of *T. persicum* were fragrant and yellow, but the essential oil (EO) was more fragrant and Arak (AR) was dark yellow.

The values of densities for EO and AR were 0.97 g cm^-3^ and 1.09 g cm^-3^, respectively*. *No previous studies were reported in this parameter with which a comparison could be made with the results of our present analysis.

The amount of essential oil isolated from the aerial parts of *T. persicum* through the steam distillation was 1.00 g/100 g dry-weight plant (DW) and the productivity of AR was 0.55 g/100 g DW.

Although there are no reports about the productivity of the Arak of *T. persicum,* previous studies have shown that *Teucriums *are generally rich in essential oils, for example, the oil yields of *T. montanum* and *T. marum* were calculated as 0.47% DW and 0.59% (v/w), respectively (29, 30) and the productivity of *T. ramosissimum *Desf. was 0.14% (w/w) (8).

**Table1:** The composition of the essential oils (EO) and Arak (AR) of the aerial parts of *Teucrium persicum.*

**Compounds**	**RI**	Composition%	
		**AR**	**EO**
*α*-Thujene	922	-	0.1
*α*-Pinene	928	-	0.3
Octane,4-ethyl-	951	0.2	-
Nonane,5-methyl-	956	0.3	-
Nonane,3-methyl-	968	0.3	-
Sabinene	969	-	0.1
*β*-Pinene	971	-	0.4
*β*-Myrcene	990	0.2	1.6
Herboxide	1005	0.1	0.2
*α*-Terpinene	1013	0.1	0.1
*ρ*-Cymene	1023	T	0.2
1,8-Cineole	1032	4.1	5.7
*β*-Ocimene	1037	-	0.3
Ocimene	1048	0.1	0.5
*γ*-Terpenine	1056	-	0.1
Linalool oxide	1073	3.5	1.2
*α*-Terpinolene	1086	0.1	0.3
Linalool	1109	10.4	7.6
Oct-1-en-3-yl acetate	1112	0.7	0.6
2-Menthenol	1123	0.1	0.1
1,3,8-p-Menthatriene	1130	t	0.2
Sabinol	1136	0.1	0.1
trans-Pinocarveol	1139	0.1	t
Nerol oxide	1156	0.4	0.4
Borneol	1169	0.8	0.6
Terpinene-4-ol	1178	0.5	0.3
*γ*-Terpineol	1198	7.3	4.4
Myrtenol	1200	-	0.1
3,7-Octadiene-2,6-diol,2,6-dimetyl-	1207	0.1	-
3,5,7-Octatriene-2-ol,2,6-dimetyl-	1213	-	0.1
trans-Carveol	1215	0.3	0.2
cis-Carveol	1225	0.3	0.4
2-Oxabicyclo[2.2.2]octan-6-ol, 1,3,3-trimethyl-	1228	0.1	-
Nerol	1235	0.7	0.6
Linalyl acetate	1262	5.6	7.7
Caprinic alcohol	1278	0.4	0.6
Carvacrol	1326	t	0.3
*γ*-Elemene	1337	0.5	0.9
Piperitenone	1345	-	0.1
*α*-Terpinyl acetate	1356	6.7	7.9
Carvyl acetate	1367	0.9	1.1
*α*-Copaene	1377	-	0.1
Geranyl acetate	1388	4.6	2.4
*β*-Elemene	1393	0.4	-
*α*-Elemene	1394	0.2	1.2
*α*-Gurjunene	1409	0.3	0.5
*β*-Caryophyllene	1420	0.2	0.5
Aromadendrene	1442	1.0	1.4
4(14),5-Muuroladiene	1447	t	0.2
*α*-Humulene	1453	t	0.2
*γ*-Gurjunene (5,11-Guaiadiene)	1459	0.3	0.6
Dodecyl alcohol	1473	0.3	-
Cadina-1(6),4-diene	1473	-	0.2
Germacrene-D	1481	0.2	0.7
*β*-Selinene	1487	0.3	0.7
*δ*-Cadinene	1492	0.2	0.3
Bcyclogermacrene	1499	1.2	2.3
*α*-Murrolene	1502	0.8	1.1
*γ*- Cadinene	1517	0.6	1.2
Phenol, 2,4-bis(1,1-dimethylethyl)-	1519	0.5	0.4
1,4-Cadinadiene	1532	6.5	9.2
*α*-Cadinene	1535	7.5	9.7
Cadina-1(2),4-diene	1537	-	0.3
Calamenene	1541	0.1	0.2
*α*-Calacorene	1545	0.2	0.1
Palustrol	1570	-	0.1
Viridiflorol	1592	0.3	0.4
Caryophyllene oxide	1595	-	0.1
Ledol	1607	t	0.2
*β*-Oplopenone	1611	t	0.1
1,10-di-epi-Cubenol	1619	t	0.2
*γ*-Eudesmol	1623	0.5	0.7
*α*-Cadinol	1650	4.4	2.9
*β*-Eudesmol	1661	1.7	2.1
Cadinol	1669	6.3	6.2
Buchariol	1683	-	t
*α*-Bisabolol	1684	0.2	0.3
Acorenone B	1703	2.1	2.5
Aromadendrene oxide	1748	-	0.1
Spathulenol	1784	1.8	0.3
Hexadecanol	1882	-	0.2
Phytol	1951	t	0.1
Manoyl oxide	2011	t	0.2
Octadecanal	2038	t	t
Geranyl 3-phenylpropanoate	2135	t	t
Geranyl linalool	2192	t	t
Grouped compounds:			
Monoterpene hydrocarbons		1.2	3.9
Oxygen-containing monoterpenes		48.4	42.9
Sesquiterpene hydrocarbons		20.4	31.8
Oxygen-containing sesquiterpenes		17.2	16.2
Miscellaneous		0.7	0.9
Total identified compounds		88.5	95.8

RI, Retention Indices relative to C8-C24 *n*-alkanes on the HP-5MS column.Components listed in the order of elution from a HP-5MS column. t: trace (< 0.05%)

*Chemical composition of essential oil and Arak*

In total, 88 components were identified in the essential oil and Arak of *T. persicum.* Most of these compounds have already been reported in the essential oils of *Teucrium *species (6, 29, 31-36). Table 1 shows the components, retention indices and percentage of composition. They are listed in the order of their elution from a HP-5MS column. Seventy-nine compounds were identified in EO representing 95.8% of total oils. As shown in Table 1, the principal components of EO were *α*-cadinene (9.7%), 1,4-cadinadiene (9.2%), *α*-terpinyl acetate (7.9%), linalyl acetate (7.7%), and linalool (7.4%). The major parts of EO were sesquiterpenes (48.0%). In this fraction, sesquiterpene hydrocarbons (31.8%) were prevailing. Monoterpenes determined 46.9% of the oil, with a prevalence of oxygen-containing monoterpenes (43.0%).

In AR, 70 compounds were identified as representing 88.54% of the oil. The major constituents in the AR were determined to be linalool (10.4%), *α*-cadinene (7.5%), *γ*-terpineol (7.3%), *α*-terpinyl acetate (6.6%), 1,4-cadinadiene (6.5%), cadinol (6.3%), and linalyl acetate (5.6%). Monoterpenes formed the most abundant portion of AR (49.6%), with a predominance of oxygen-monoterpenes (48.4%), while monoterpene hydrocarbon was only 1.1%. Overall, 37.6% of the oil consisted of sesquiterpenes, of which oxygen-containing sesquiterpenes were 17.2%.

As shown in Table 1, most compounds identified from different oils were almost the same, but the amounts of corresponding components were different. According to data, the main groups of component in the EO are cadinane-sesquiterpenes, especially *α*-cadinene and 1, 4-cadinadiene, but in AR, the major constituents are acyclic monoterpenes, mainly linalool.

So far, more than 200 cadinane-type sesquiterpenes are known. They show various biological activities, such as wood preservative, fungicide, anti-malaria and anti-HIV (36, 37, 40). In all oils, there are small amounts of diterpenoids such as geranyl linalool, manoyl oxide, and phytol. Hydrolat volatile compositions have been the subject of limited research and there are no earlier studies about the chemical components of Arak with which to compare our results. The major components in hydrolat usually had the same corresponding essential oil ingredients (18).

When the chemical profile of the essential oil was compared with earlier studies, the results were somewhat different. Masoudi *et al.* (2009) identified 31 constituents corresponding to 95.9% of the total in the oil and also reported epi-*α*-cadinol (23.2%) as the major component (37). In addition, Javidnia *et al*. (2007) reported 81 compounds, representing 93.5% of the total oil, and the major compounds were caryophyllene oxide (10.6%), *α*-pinene (9.4%), geranyl linalool (7.8%), *γ*-cadinene (7.4%), elemol (6.9%), and *α*-cadinol (5.5%).

Differences in oil compounds may be influenced with geographical differences, time of plant harvesting and preparation process (4, 30, 37).

In contrast to our study, earlier investigations indicated that sesquiterpenes were the major compounds in the essential oil of *Teucriums*. For example, 57 components were recognized in the oil of *T. alopecurus* and predominant compounds were sesquiterpene hydrocarbons (61.3%) and oxygenated sesquiterpenes (26.9%) (8). Vukovic *et al.* (2007) studied the essential oil of *T. montanum* and recognized 45 compounds, representing 97.95% of the total; the main constituents of the oil were mono and sesquiterpenes hydrocarbons (30). Saroglou *et al*. (2007) studied the oil of *T. royleanum* and reported that the sesquiterpene hydrocarbons formed the main part (42.2%) of the oil (32), but in *T. persicum,* the prevailing compounds were oxygenated monoterpenes (45.7% ± 3.8%) followed by sesquiterpene hydrocarbons (26.1% ± 8.08%).

In the phytochemical investigation of *T. persicum*, buchariol, a sesquiterpenoid guaiane skeleton type (4,10-epoxy-6 *α*-hydroxyguaiane), was obtained. Its structure was elucidated with the help of Mass, IR and NMR spectroscopy including 1D and 2D experiments. It was confirmed to compare with articles (38, 39).

The obtained mass spectrum was compared to GC/MS chromatogram. The relevant peak was found as trace. To the best of our knowledge, this is the first time that this compound is introduced in the essential oil.

Its Kovats index was 1683, in HP-5MS column and the above mentioned temperature program. The mass fragmentation (EIMS) was: m/z 238.4 (8.3%), 221.4 (5.6%), 203 (6.7%), 195 (7.4%), 43 (100%).

*Total phenolic content*

Phenolic components are one of the secondary metabolites in plants that, due to their redox properties, are considered as the antioxidants (22, 28). As shown in Table 2, The EO revealed a higher content of total phenolics than the AR (1.71 ± 0.12 mg GAE/g DW and 1.36 ± 0.11 mg GAE/g DW, respectively). The phenolic content of the samples that we analyzed is less than the values found for polar and non-polar extracts of *T. *c*hamaedrys* (97.12 ± 1.28 and 69.75 ± 2.62 μg GAE/mg, respectively). However, EO and AR showed relatively lower levels of phenolic content compared with *T. arduini* flowers (30.49 ± 1.00 mg GAE/g DW) and leaves (23.39 ± 3.60 mg GAE/g DW) (2, 40).

*Antioxidant activity*

*Ferric reducing antioxidant power assay (FRAP assay)*

The reducing power of the oils of *T. persicum* measured under the FRAP assay and an aqueous solution of ferrous sulphate (50-500 µmol/mL, y = 0.002x - 0.025, R² = 0.993) was used as a calibration curve. The results were expressed as µmol Fe^2+^ equivalent per g of dry-weight plant (DW). In Table 2, the FRAP value points to a considerably higher reducing power of EO (220 ± 7.2 µmol Fe^2+^/g DW) compared with that of AR (113 ± 5.4 µmol Fe^2+^/g DW). The results of our study compared with the work of Šamec *et al*. (2010) indicate that the reducing powers of EO and AR are greater than the reducing power of the average FRAP value for the leaf (75.81 ± 34.99 µmol Fe^2+^/g DW) and flower (97.65 ± 54.38 µmol Fe^2+^/g DW) infusions of *T. arduini* (2). The results are compatible with those of TPC.

*DPPH radical scavenging activity*

In the DPPH assay, the radical scavenging activity of the samples was compared to BHA and α-tocopherol as the standards. EO exhibited higher radical scavenging potential (IC_50_= 0.29 mg/mL) than AR (IC_50_= 4.19 mg/mL). These samples were less effective than BHA (IC_50_= 0.016 mg/mL) and *α*-tocopherol (IC_50_=0.015 mg/mL).

Kadifkova Panovsk *et al.* (2005) reported that the extract of *T. polium*, *T. chamaedrys* and *T. montanum* possessed DPPH radical scavenging activities with IC_50_ of 10, 11, and 10 mg/mL, respectively. They compared these results to the standard components, silymarin, quercetin, and luteolin, with IC_50_: 1.96, 0.06, and 0.08 mg/mL, respectively , and found the *Teucrium* extracts to be less effective than standards (41).

These results show that *Teucrium* is a good antioxidant agent, but the oils of *T. persicum* are more effective than the extracts of *T. polium*, *T. chamaedrys,* and *T. montanum*. The present assay confirms the values obtained from TPC and FRAP.

**Table 2:** Antioxidant activity and Total phenolic content (TPC) of essential oil (EO) and Arak (AR) of aerial part of *Teucrium persicum*

TPC (mg GAE^*****^/g DW)	RP assay^***^ µg BHA/g DW^****^	FRAP assay^**^ µmol Fe^2+^ /g DW	DPPH assay^*^ mg/mL	
1.36 ± 0.11	34.1 ± 2.7	113 ± 5.4	4.19	**AR**
1.71 ± 0.12	51.7 ± 4.3	220 ± 7.2	0.29	**EO**

Values are in the mean ± SD, n = 3 for each experiment.^*^DPPH radical scavenging assay. ^**^Ferric reducing power assay.^***^Reducing power assay.^****^Dry-weight.^*****^Gallic acid equivalents.

*Reducing power*

BHA was used as the standard calibration curve (5- 60 µg/mL, y = 0.006x + 0.058, R² = 0.997). The reducing power of EO (51.7 ± 4.3 µg BHA/g DW) was higher than that of AR (34.1 ± 2.7 µg BHA/g DW). The results of FRAP, DPPH, and TPC are compatible with the reducing power values.

Finally, the results show that the oils analyzed in our study revealed good antioxidant activity and high yields, so they could be suggested for use as natural antioxidants in food and cosmetic products.
